# Associations of immunological factors with metabolic syndrome and its characteristic elements in Chinese centenarians

**DOI:** 10.1186/s12967-018-1691-4

**Published:** 2018-11-19

**Authors:** Shihui Fu, Yao Yao, Faqin Lv, Fu Zhang, Yali Zhao, Fuxin Luan

**Affiliations:** 1 Department of Geriatric Cardiology, Chinese People’s Liberation Army General Hospital, Beijing, China; 2 Department of Cardiology and Hainan Branch, Chinese People’s Liberation Army General Hospital, Beijing, China; 3 Institute of Geriatrics, Chinese People’s Liberation Army General Hospital, Beijing, China; 4 Beijing Key Laboratory of Aging and Geriatrics, Chinese People’s Liberation Army General Hospital, Beijing, China; 5 Department of Ultrasound, Hainan Branch of Chinese People’s Liberation Army General Hospital, Sanya, China; 6 Central Laboratory, Hainan Branch of Chinese People’s Liberation Army General Hospital, Sanya, China

**Keywords:** Abdominal obesity, Chinese centenarians, Complement C3, Immunoglobulin E, Metabolic syndrome

## Abstract

**Background:**

Metabolic syndrome (MetS) has an increased prevalence (approximately 20–25% of the adult population) all over the world. Immunological function is significantly associated with the development of MetS, and MetS is beginning to be considered as a chronic immune-related disease. The present study addressed on the associations of immunological factors with MetS and its characteristic elements in Chinese centenarians.

**Methods:**

Hainan is a longevity region with the highest population density of centenarians in China. The China Hainan Centenarian Cohort Study has a considerable sample size, and provides a significant population-based sample of centenarians. Home interview, physical examination and blood analysis were conducted following standard procedures.

**Results:**

All centenarians had a median age of 102 (100–115) years, and the proportion of females was 80.8%. The proportion of centenarians with MetS was 16.0% (135 centenarians). Abdominal obesity, hypertension, dyslipidemia and diabetes mellitus had a prevalence of 26.4% (223 centenarians), 73.7% (623 centenarians), 40.4% (341 centenarians) and 10.7% (90 centenarians), respectively. In Logistic regression analyses, MetS was significantly associated with immunoglobulin E and complement C3 levels (P < 0.05 for all). Abdominal obesity was significantly associated with immunoglobulin E and complement C3 levels (P < 0.05 for all).

**Conclusions:**

The present study provides epidemiological evidence that MetS has significant associations with immunoglobulin E and complement C3 levels, and demonstrates that abdominal obesity is significantly associated with immunoglobulin E and complement C3 levels in Chinese centenarians.

## Background

Metabolic syndrome (MetS) has an increased prevalence (approximately 20–25% of the adult population) all over the world [1, 2]. MetS is a risk factor for the development of cardiovascular disorders, and results in an increased risk of cardiovascular events and all-cause mortality [3, 4]. Characteristic elements of MetS include abdominal obesity, high blood pressure, high blood glucose and abnormal blood lipids, all of which are risk factors for cardiovascular disorders and mortality [5]. Immunological function is significantly associated with the development of MetS, and MetS is beginning to be considered as a chronic immune-related disease [6]. It has been suggested that elevated immunological factors, such as immunoglobulin A, E, G, M, kappa and lambda, and complement C3 and C4, correlate with cardiovascular mortality [7]. Regarding the associations of immunological factors with MetS and its characteristic elements, scarce studies have been performed and obtained contradicting results, making further epidemiological investigations necessary [3, 8–10].

The prevalence of MetS increases with age, approaching 42.0% in US adults aged 70 years or more [11]. Epidemiological studies of MetS in the elderly, particularly in the centenarians, are therefore of great value. The centenarians have been suggested to have a delayed or escaped onset and interaction of age-related abnormalities [12]. Some centenarians may experience a delayed onset of age-related abnormalities (delayers), while others may do not succumb to any age-related abnormalities (escapers) [13]. Thus, the centenarians may represent a prototype of successful aging [14]. However, it is still under scientific debate [15]. More importantly, what is this model of successful aging? Studies analyzing this model in the centenarians could provide valuable information for early promoting successful aging and preventing age-related abnormalities. As a possible part of this model, whether the interaction between MetS and immunological factors exists in the aging process of centenarians is still unclear and needs further studies.

Although the associations of immunological factors with MetS and its characteristic elements have already been explored in previous studies, they have not been done in the centenarians, particularly in Chinese centenarians, in a large-scale study [8, 9]. Consequently, there is a need to carry out the investigations in Chinese centenarians. Hainan is a longevity region with the highest population density of centenarians in China. The China Hainan Centenarian Cohort Study (CHCCS) has a considerable sample size, and provides a significant population-based sample of Chinese centenarians. The present study addressed on the associations of immunological factors with MetS and its characteristic elements in Chinese centenarians.

## Methods

### Study population

The CHCCS involves a population-based cohort of individuals aged 100 years or more recruited between July 2014 and December 2016 in 18 cities and counties of Hainan Province, China. Its cohort profile has been described previously [16]. Sample size (1002 centenarians) was determined by the National Civil Registry and provided by the Hainan Civil Affairs Bureau. Age was consistent with national identification cards. The final analysis had 845 centenarians with complete data. All these centenarians had no diagnosed rheumatoid arthritis and other autoimmune diseases.

### Standard procedures

Home interview, physical examination and blood analysis were conducted following standard procedures by the same research team and central laboratory of the Hainan Branch of Chinese People’s Liberation Army General Hospital [17]. The research team included internists, geriatricians, cardiologists, endocrinologists, nephrologists and nurses. Based on the recommendations by the World Health Organization, waist circumference (WC) was measured twice using the tapes at the midpoint between the last rib and the iliac crest [18]. Systolic and diastolic blood pressures (SBP and DBP) were measured in the right arm of seated centenarians. Samples of venous blood were extracted from the centenarians and transported within 4 h in chilled bio-transport containers (4 °C) to our central laboratory. Serum levels of immunoglobulins and complements were measured with a fully automatic protein analyzer (BNII; Siemens AG, Munich, Germany; CV within group: 1.4–4.7%; CV between groups: 2.5–5.5%). Serum levels of triglyceride, high-density lipoprotein cholesterol (HDL-C) and fasting blood glucose (FBG) were measured by enzymatic assays (Roche Products Ltd, Basel, Switzerland) with a fully automatic biochemical autoanalyzer (Cobas c702; Roche Products Ltd, Basel, Switzerland; CV within group: 0.5–2.3%; CV between groups: 0.8–3.1%) and low-density lipoprotein cholesterol (LDL-c) was calculated. All assays were conducted by qualified technicians without knowledge of the clinical data.

### Variable definitions

According to the worldwide consensus on the definition of MetS established by the International Diabetes Federation, the centenarians were considered to have MetS if they had abdominal obesity and at least two of the following factors were satisfied: high blood pressure (SBP ≥ 130 mmHg or DBP ≥ 85 mmHg or previously diagnosed hypertension), high blood glucose (FBG ≥ 5.6 mmol/L or previously diagnosed diabetes mellitus), and abnormal blood lipids (triglyceride ≥ 1.7 mmol/L or HDL-C < 1.0 mmol/L in males and < 1.3 mmol/L in females or previously diagnosed dyslipidemia) [19]. Based on Chinese guidelines on the prevention and control of obesity, the presence of abdominal obesity was established when WC ≥ 85 cm for men and ≥ 80 cm for women [20]. Hypertension was defined as SBP ≥ 140 mmHg, DBP ≥ 90 mmHg or using anti-hypertensive drugs [21]. Diabetes mellitus was defined as FBG ≥ 7.0 mmol/L or using anti-diabetic drugs/insulin [22]. Dyslipidemia was defined as triglyceride ≥ 1.7 mmol/L, LDL-C ≥ 3.37 mmol/L, HDL-C ≥ 1.04 mmol/L or using lipid-regulating drugs [23].

### Statistical analyses

Continuous variables with a normal distribution were presented as mean ± standard deviation, and then compared with a Student’s t-test. Continuous variables with a skewed distribution were presented as median (interquartile range), and then compared with a Mann–Whitney U-test. Categorical variables were presented as number (percentage), and then compared with a Chi square test. Logistic regression models were carried out with and without the adjustment of age and sex to assess the associations of immunological factors with MetS and its characteristic elements. MetS, its characteristic elements including abdominal obesity, high blood pressure, high blood glucose and abnormal blood lipids, and different morbidities including hypertension, diabetes mellitus and dyslipidemia were used as dependent variables in these models. Immunological factors, such as immunoglobulin A, E, G, M, kappa and lambda, and complement C3 and C4, were used as independent variables in these models. Statistical significance was assessed using 2-tailed p values at a threshold of 0.05. Statistical analyses were carried out using SPSS version 17 (SPSS Inc., Chicago, IL, US).

## Results

**Fig. 1:**
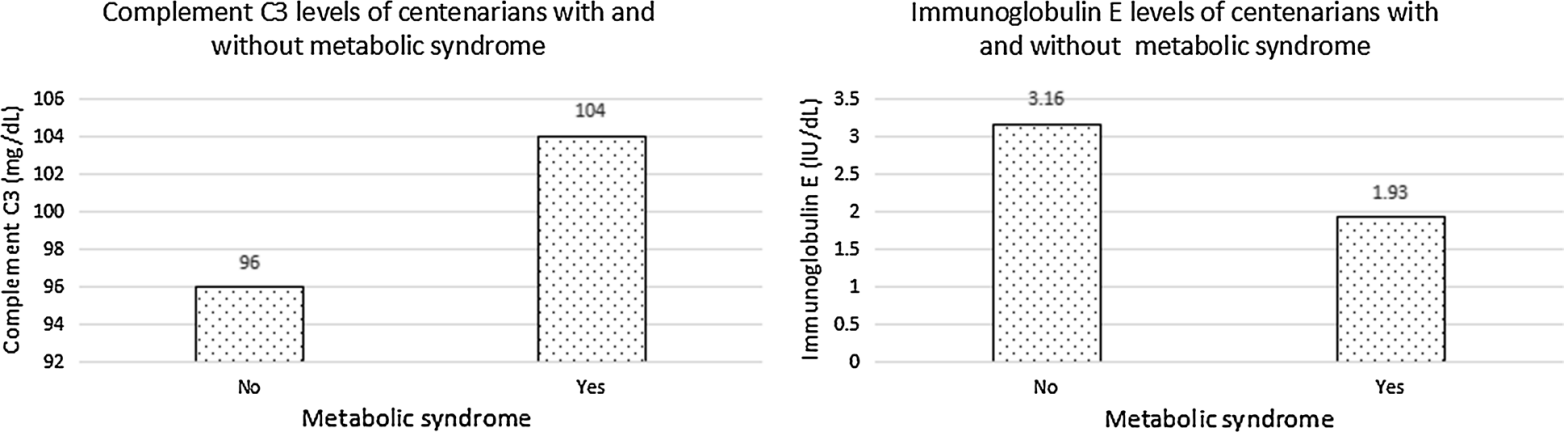
Complement C3 and immunoglobulin E levels of centenarians with and without metabolic syndrome

All centenarians had a median age of 102 (100–115) years, and the proportion of females was 80.8%. The proportion of centenarians with MetS was 16.0% (135 centenarians). Abdominal obesity, hypertension, dyslipidemia and diabetes mellitus had a prevalence of 26.4% (223 centenarians), 73.7% (623 centenarians), 40.4% (341 centenarians) and 10.7% (90 centenarians), respectively. Table 1 provides the features of all centenarians with and without MetS. There were more females, higher prevalence of abdominal obesity, hypertension, dyslipidemia and diabetes mellitus, higher levels of WC, SBP, DBP, triglyceride and LDL-C, and lower levels of HDL-C in the centenarians with MetS than those without MetS (P < 0.05 for all). Meanwhile, the centenarians with MetS had lower levels of immunoglobulin E and higher levels of complement C3 than those without MetS (P < 0.05 for all; Fig. 1).

**Table 1:** Features of all centenarians with and without MetS

Features	All	Non-MetS (n = 710)	MetS (n = 135)	P value
Age (years)^a^	102 (101–104)	102 (101–104)	102 (101–104)	0.268
Females (%)^b^	683 (80.8)	558 (78.6)	125 (92.6)	< 0.001
Abdominal obesity (%)^b^	223 (26.4)	88 (12.4)	135 (100.0)	< 0.001
Hypertension (%)^b^	623 (73.7)	508 (71.5)	115 (85.2)	0.001
High blood pressure (%)^b^	712 (84.3)	586 (82.5)	126 (93.3)	0.002
Dyslipidemia (%)^b^	341 (40.4)	248 (34.9)	93 (68.9)	< 0.001
Abnormal blood lipids (%)^b^	317 (37.5)	212 (29.9)	105 (77.8)	< 0.001
Diabetes mellitus (%)^b^	90 (10.7)	59 (8.3)	31 (23.0)	< 0.001
High blood glucose (%)^b^	247 (29.2)	172 (24.2)	75 (55.6)	< 0.001
WC (cm)^a^	75 (70–81)	74 (68–78)	85 (82–90)	< 0.001
SBP (mmHg)^a^	150 (136–170)	148 (133–168)	158 (145–179)	< 0.001
DBP (mmHg)^a^	76 (67–83)	75 (66–83)	78 (72–86)	0.002
Triglyceride (mmol/L)^a^	1.03 (0.80–1.41)	0.98 (0.77–1.30)	1.57 (1.02–1.90)	< 0.001
HDL-C (mmol/L)^a^	1.40 (1.17–1.67)	1.43 (1.21–1.71)	1.22 (1.02–1.45)	< 0.001
LDL-C (mmol/L)^a^	2.72 (2.27–3.26)	2.70 (2.26–3.23)	2.85 (2.44–3.42)	0.015
FBG (mmol/L)^a^	4.83 (4.20–5.76)	4.73 (4.15–5.55)	5.74 (4.56–6.77)	< 0.001
Immunoglobulin A (mg/dL)^a^	336 (253–430)	336 (252–429)	338 (256–436)	0.367
Immunoglobulin G (mg/dL)^a^	1580 (1360–1830)	1590 (1360–1853)	1540 (1380–1760)	0.184
Immunoglobulin M (mg/dL)^a^	101 (71–140)	102 (71–141)	97 (71–135)	0.320
Immunoglobulin E (IU/dL)^a^	2.96 (0.88–8.77)	3.16 (0.97–9.48)	1.93 (0.64–5.69)	0.002
Immunoglobulin kappa (mg/dL)^a^	414 (355–484)	417 (355–490)	407 (355–458)	0.096
Immunoglobulin lambda (mg/dL)^a^	209 (177–251)	210 (178–252)	205 (173–242)	0.280
Complement C3 (mg/dL)^a^	97 (85–111)	96 (84–109)	104 (92–122)	< 0.001
Complement C4 (mg/dL)^a^	23 (18–28)	23 (18–27)	24 (20–29)	0.085

*MetS* metabolic syndrome, *WC* waist circumference, *SBP* systolic blood pressure, *DBP* diastolic blood pressure, *HDL-C* high-density lipoprotein cholesterol, *LDL-C* low-density lipoprotein cholesterol, *FBG* fasting blood glucose

^a^Described as median (interquartile range), and then compared with a Mann–Whitney U test

^b^Described as number (percentage), and then compared with a Chi square test

In Logistic regression analyses (Table 2), MetS was significantly associated with immunoglobulin E {crude odds ratio (OR) [95% confidence interval (95% CI)] 0.980 (0.964–0.996); adjusted OR (95% CI) 0.981 (0.966–0.997); P < 0.05 for all} and complement C3 levels [crude OR (95% CI) 1.022 (1.013–1.030); adjusted OR (95% CI) 1.020 (1.012–1.029); P < 0.05 for all]. As shown in Table 3, abdominal obesity was significantly associated with immunoglobulin E [crude OR (95% CI) 0.987 (0.976–0.997); adjusted OR (95% CI) 0.987 (0.977–0.998); P < 0.05 for all] and complement C3 levels [crude OR (95% CI) 1.018 (1.011–1.026); adjusted OR (95% CI) 1.018 (1.010–1.025); P < 0.05 for all]. Additionally, dyslipidemia [crude OR (95% CI) 1.034 (1.026–1.042); adjusted OR (95% CI) 1.034 (1.026–1.042); P < 0.05 for all] and abnormal blood lipids [crude OR (95% CI) 1.021 (1.014–1.028); adjusted OR (95% CI) 1.020 (1.013–1.027); P < 0.05 for all] were significantly associated with complement C3 levels.

**Table 2:** Associations of immunological factors with metabolic syndrome

Features	OR	95% CI	P value	OR	95% CI	P value
Metabolic syndrome						
Immunoglobulin A (mg/dL)	1.000	0.999–1.001	0.796	1.000	0.999–1.001	0.741
Immunoglobulin G (mg/dL)	1.000	0.999–1.000	0.076	1.000	0.999–1.000	0.120
Immunoglobulin M (mg/dL)	1.000	0.997–1.003	0.877	0.999	0.996–1.002	0.648
Immunoglobulin E (IU/dL)	0.980	0.964–0.996	0.016	0.981	0.966–0.997	0.021
Immunoglobulin kappa (mg/dL)	0.998	0.997–1.000	0.066	0.999	0.997–1.000	0.104
Immunoglobulin lambda (mg/dL)	0.998	0.995–1.001	0.315	0.999	0.996–1.002	0.356
Complement C3 (mg/dL)	1.022	1.013–1.030	< 0.001	1.020	1.012–1.029	< 0.001
Complement C4 (mg/dL)	1.017	0.997–1.038	0.097	1.016	0.996–1.036	0.128

*OR* odds ratio, *95% CI* 95% confidence interval

**Table 3:** Associations of immunological factors with characteristic elements of metabolic syndrome

Features	OR	95% CI	P value	OR	95% CI	P value
Abdominal obesity						
Immunoglobulin E (IU/dL)	0.987	0.976–0.997	0.015	0.987	0.977–0.998	0.019
Complement C3 (mg/dL)	1.018	1.011–1.026	< 0.001	1.018	1.010–1.025	< 0.001
Hypertension						
Immunoglobulin E (IU/dL)	0.996	0.991–1.001	0.098	0.996	0.991–1.001	0.099
Complement C3 (mg/dL)	1.007	1.000–1.014	0.056	1.006	0.999–1.014	0.084
High blood pressure						
Immunoglobulin E (IU/dL)	0.995	0.990–1.001	0.086	0.995	0.990–1.001	0.086
Complement C3 (mg/dL)	1.008	1.000–1.017	0.060	1.008	0.999–1.017	0.074
Dyslipidemia						
Immunoglobulin E (IU/dL)	0.995	0.989–1.001	0.132	0.995	0.989–1.002	0.137
Complement C3 (mg/dL)	1.034	1.026–1.042	< 0.001	1.034	1.026–1.042	< 0.001
Abnormal blood lipids						
Immunoglobulin E (IU/dL)	0.998	0.992–1.003	0.411	0.998	0.992–1.003	0.433
Complement C3 (mg/dL)	1.021	1.014–1.028	< 0.001	1.020	1.013–1.027	< 0.001
Diabetes mellitus						
Immunoglobulin E (IU/dL)	1.000	0.992–1.008	0.922	1.000	0.992–1.008	0.915
Complement C3 (mg/dL)	0.994	0.984–1.004	0.262	0.994	0.984–1.004	0.254
High blood glucose						
Immunoglobulin E (IU/dL)	0.997	0.991–1.004	0.376	0.997	0.991–1.003	0.373
Complement C3 (mg/dL)	0.997	0.990–1.004	0.387	0.997	0.990–1.004	0.387

*OR* odds ratio, *95% CI* 95% confidence interval

## Discussion

As the first study in the world, the present study demonstrates that MetS has significant associations with immunoglobulin E and complement C3 levels, and abdominal obesity is significantly associated with immunoglobulin E and complement C3 levels in Chinese centenarians.

MetS is a practical method for identifying individuals with a high risk of cardiovascular disorders and all-cause mortality [24, 25]. MetS has complex pathophysiology, and its relationship with immunological function has been only partially elucidated [5]. Previous studies have found that immunological factors may be related to MetS [8, 9]. However, other studies have not observed significant associations of these immunological factors with MetS [3, 10]. Because previous studies are limited and their results are controversial, further epidemiological investigations are needed to evaluate these associations. To our knowledge, there has been scarce study assessing these associations in the elderly, and nearly no study, particularly large-scale study, investigating these associations in the centenarians [8, 9]. It is essential to make a comparison of these associations between different age-group populations. With a considerable population-based centenarian samples, the present study confirms that MetS have significant associations with immunoglobulin E and complement C3 levels in Chinese centenarians.

The complement system plays an important role in innate and adaptive immunological mechanisms. Complement C3 is a major protein of the complement pathways [26]. Complement C3 levels have been correlated with MetS in previous studies [27, 28]. There may be an increase in complement C3 levels in relation to MetS in the elderly [29, 30]. However, another study has not observed significant associations between MetS and complement C3 levels [10]. The present study finds that MetS is significantly associated with complement C3 levels in Chinese centenarians. Although the mechanism responsible for this association has not yet been known, there are some possible explanations. Complement C3 is synthesized in the liver [31]. The cytokines that stimulate hepatic production of complement C3 are mainly secreted by excess adipose tissue [30]. Complement C3 is also synthesized by activated adipocytes and macrophages, and behaves as both a cytokine and an adipokine [32]. These cytokines may promote insulin resistance by increasing the phosphorylation and proteosomal degradation of insulin receptor substrates or by affecting the insulin receptor-substrate interaction [33]. As the main degraded product and active fragment of C3, acylation stimulating protein (ASP, C3a desArg) has insulin-like properties and favors lipid synthesis in adipocytes [34]. Thus, in the same way that increased insulin levels may be induced by the presence of insulin resistance, an ASP resistance may cause an increased ASP precursor levels (C3) [35].

Immunoglobulin E levels are often associated with immunological function and allergic response, and has been reported to have significant associations with cardiovascular disorders [7]. Abnormal immunological function may contribute to the development of MetS [6]. Previous studies have proposed that among young, middle-aged and older adults, immunoglobulin E levels are significantly higher in individuals with MetS than those without MetS [9]. However, another study has not found the associations between MetS and immunoglobulin E levels [3]. Furthermore, there is a lack of evidence to show the associations between MetS and immunoglobulin E levels in the centenarians, particularly in China. The present study finds significant association between MetS and immunoglobulin E levels in Chinese centenarians. The relationships between MetS and immunoglobulin E levels are complicated, and the specific mechanism remains unclear. In experimental animals, allergic response and immunoglobulin E are suppressed by insulin resistance [36]. Immunoglobulin E not only induces the activation and apoptosis of endothelial cells, mast cells and macrophages, but also affects the expression and function of cell-related cytokines [37]. These cells and cytokines have been shown to be very significant in the development of obesity and MetS [38]. Thus, immunoglobulin E may be involved in MetS by directly or indirectly interacting with these cells and cytokines [36].

Previous studies have shown that complement C3 and immunoglobulin E levels have strong associations with insulin resistance and abdominal obesity in the young and middle-aged populations [39–42]. Complement C3 and immunoglobulin E levels have been strongly associated with insulin resistance and abdominal obesity in the elderly [30]. The present study shows abdominal obesity as an associated characteristic element of MetS with immunoglobulin E levels in Chinese centenarians. Moreover, the present study illustrates that abdominal obesity is a characteristic element of MetS associated with complement C3 levels in Chinese centenarians. Of all the characteristic elements of MetS, WC and abdominal obesity have been considered to be the most strongly associated with insulin resistance and MetS [30]. Abdominal adipose may be an immunological organ and result in insulin resistance and MetS [43, 44]. High expression of C3 is correlated with visceral adipose and abdominal obesity [38]. ASP (C3a-desArg) can stimulate lipogenesis in adipose cells. Thus, complement C3 may aggravate abdominal obesity and affect lipid metabolism, further contributing to the development of MetS [45, 46]. Adipose tissue may produce complement C3 and trigger an immunological response [43].

The present study had one limitation. It had no interpretation of these associations between immunological factors and MetS in form of external factors, such as dietary and environmental factors, or endogenous factors, such as hormones or adipokines.

## Conclusions

The present study provides epidemiological evidence that MetS has significant associations with immunoglobulin E and complement C3 levels in Chinese centenarians. Moreover, the present study demonstrates that abdominal obesity is significantly associated with immunoglobulin E and complement C3 levels in Chinese centenarians.
